# Serum Protein KNG1, APOC3, and PON1 as Potential Biomarkers for Yin-Deficiency-Heat Syndrome

**DOI:** 10.1155/2016/5176731

**Published:** 2016-10-24

**Authors:** Changming Liu, Liangen Mao, Zepeng Ping, Tingting Jiang, Chong Wang, Zhongliang Chen, Zhongjie Li, Jicheng Li

**Affiliations:** Institute of Cell Biology, Zhejiang University, Hangzhou 310058, China

## Abstract

Yin-deficiency-heat (YDH) syndrome is a concept in Traditional Chinese Medicine (TCM) for describing subhealth status. However, there are few efficient diagnostic methods available for confirming YDH syndrome. To explore the novel method for diagnosing YDH syndrome, we applied iTRAQ to observe the serum protein profiles in YDH syndrome rats and confirmed protein levels by ELISA. A total of 92 differentially expressed proteins (63 upregulated proteins and 29 downregulated proteins), which were mainly involved in complement and coagulation cascades and glucose metabolism pathway, were identified by the proteomic experiments. Kininogen 1 (KNG1) was significantly increased (*p* < 0.0001), while apolipoprotein C-III (APOC3, *p* < 0.005) and paraoxonase 1 (PON1, *p* < 0.001) were significantly decreased in the serum of YDH syndrome rats. The combination of KNG1, APOC3, and PON1 constituted a diagnostic model with 100.0% sensitivity and 85.0% specificity. The results indicated that KNG1, APOC3, and PON1 may act as potential biomarkers for diagnosing YDH syndrome. KNG1 may regulate cytokines and chemokines release in YDH syndrome, and the low levels of PON1 and APOC3 may increase oxidative stress and lipolysis in YDH syndrome, respectively. Our work provides a novel method for YDH syndrome diagnosis and also provides valuable experimental basis to understand the molecular mechanism of YDH syndrome.

## 1. Introduction

Yin-deficiency-heat (YDH) syndrome, also known as the pathological condition “internal heat due to Yin deficiency,” is a common subhealth status in Traditional Chinese Medicine (TCM). YDH syndrome occurs frequently in individuals with Yin deficiency constitution, which is the fourth most common pathological constitution in general population [1]. Nowadays, YDH syndrome presents a great challenge in China, prevailing especially among white collar workers and college students [2]. YDH syndrome among individuals aged between 15 and 34 years has been shown to be more common than among other age groups [1]. Individuals with YDH syndrome present with deterioration in physical function, fatigue, weakness, emaciation, five-center (the palms, soles, and chest) heat, and tidal fever, which negatively affect the quality of life and the work productivity.

YDH syndrome could be relieved by Chinese herbal compound, and if diagnosed and treated promptly, it can be prevented from developing into disease. However, as a subhealth status without any obvious manifestations, the diagnosis of YDH syndrome has always been a tough problem. Currently, the traditional clinical diagnosis of YDH syndrome is based on subjective observation, which depends heavily on the clinical experience or knowledge of practitioner in TCM. In addition, questionnaires are commonly used to identify YDH syndrome, whereas the questions which are ambiguous or difficult-to-distinguish may lead to the bias in the test. The current methods for the diagnosis of YDH syndrome are lack of scientific basis. Therefore, novel methods suitable for YDH syndrome diagnosis are urgently needed.

Given the noninvasiveness and easy accessibility, serum is frequently used to screen diagnostic biomarkers for many diseases. Furthermore, serum contains approximately 10,000 proteins from cells and tissues [3], and the alteration of protein level sensitively reflects different diseases or pathological conditions. Former studies have successfully identified serum biomarkers for the diagnosis of breast carcinoma [4], colorectal cancer hepatic metastasis [5], and pathological characteristics of pulmonary TB with TCM syndromes [6]. In this study, we applied iTRAQ labeling coupled with two-dimensional liquid chromatography-tandem mass spectrometry (2D LCMS/MS) to investigate differentially expressed proteins in the serum of YDH syndrome rats. Further bioinformatics analysis revealed that differentially expressed proteins KNG1, APOC3, and PON1 were closely related to YDH syndrome and may act as potential biomarkers for diagnosis of YDH syndrome. Our results suggest the potential role of KNG1, APOC3, and PON1 in the diagnosis of YDH syndrome and provide important insights to understand the molecular mechanism of YDH syndrome.

## 2. Materials and Methods

### 2.1. Experimental Design

**Figure 1:**
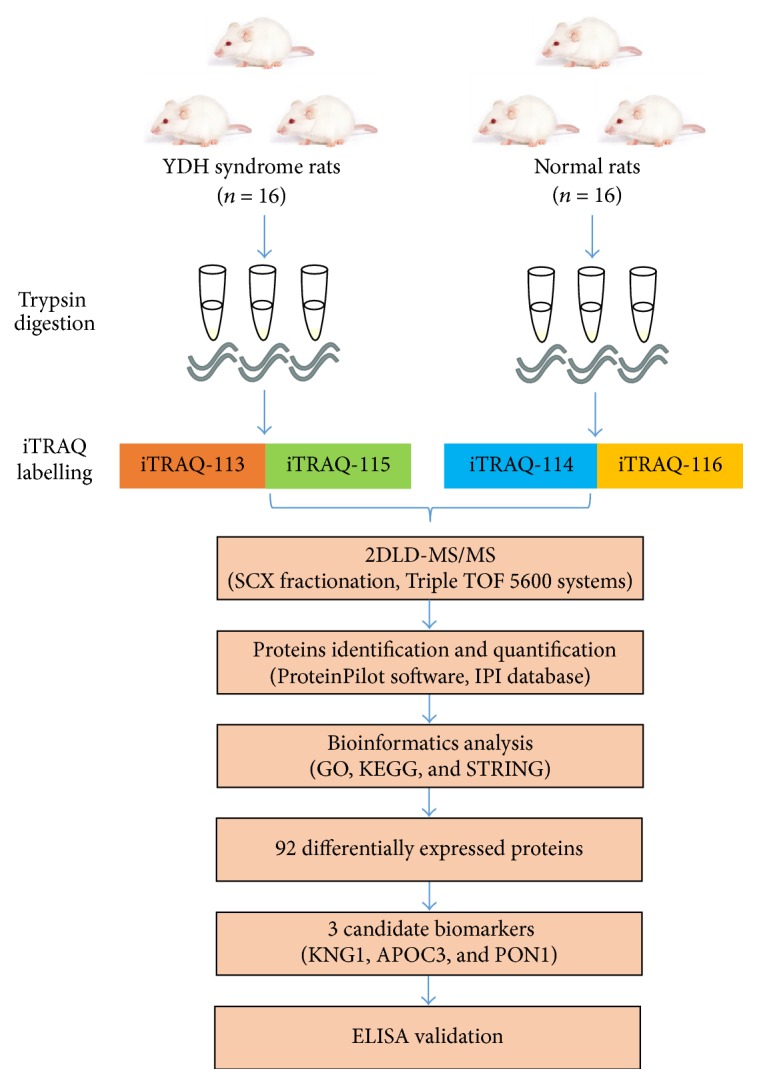
Schematic of the workflow design based on iTRAQ-2DLC-MS/MS analysis of YDH-related proteins in rats.

The schematic of the experimental workflow is shown in Figure 1. Serum samples from YDH syndrome rats and normal rats were subjected to iTRAQ-coupled 2D LC-MS/MS analysis. Bioinformatics approaches were used to explore the function of differentially expressed proteins. The expression levels of proteins were measured by ELISA.

### 2.2. Animals and Treatment

SPF female Sprague-Dawley rats, weighing 180–220 g, were purchased from the Experimental Animal Center of Zhejiang Province (China). All rats were housed at a constant temperature of 23 ± 1°C and a 12 h light/dark cycle with free access to water and standard rat diet. All experimental procedures were approved by the Zhejiang University Institutional Animal Care and Use Committee. The pungent and hot Chinese herbs, Fuzi (Radix Aconiti Praeparata), Ganjiang (Rhizoma Zingiberis), and Rougui (*Cinnamomum cassia*) were purchased from the Hangzhou Hu Qingyu Drugstore (China). These herbs with warm nature consume Yin fluid, which is commonly used in TCM to induce YDH syndrome animal models [7]. Equal amounts of Fuzi, Ganjiang, and Rougui were mixed, decocted, and concentrated to a final concentration of 4 g/mL according to the previously described method [8]. After acclimation for 7 days, rats were randomly divided into the model group (herbal decoction, 2 mL/100 g/d, by gastrogavage) and the control group (sterile saline solution, 2 mL/100 g/d, by gastrogavage). All rats were sacrificed after 3 weeks of treatment, and serum samples were collected and stored at −80°C. Thymus, spleen, and adrenal gland were immediately removed and weighed. The anesthetized rats were euthanized by cervical dislocation.

### 2.3. iTRAQ-2D LC-MS/MS

Sample pooling is commonly used in proteomic studies to increase the precision and accuracy of the results [9]. Serum samples from the model group (*n* = 16) and the control group (*n* = 16) were subjected to iTRAQ labeling according to the manufacturer's instructions (Applied Biosystems, Foster city, CA, USA), and 16 samples in each group were randomly separated into two subgroups to establish two biological replicates, so 4 pooling samples were generated. To enrich the low abundant protein, Agilent MARS-14 column (Agilent Technologies, Santa Clara, CA, USA) was used to remove 14 highly abundant proteins, including albumin, IgG, antitrypsin, IgA, transferring, haptoglobin, fibrinogen, alpha 2-macroglobulin, alpha 1-acid glycoprotein, IgM, apolipoprotein AI, apolipoprotein AII, complement C3, and transthyretin, and the eluent of protein samples were quantified by the Bradford method. The protein samples (100 *μ*g) of each group were reduced, alkylated, and digested with trypsin (Sigma, St. Louis, MO, USA) overnight at 37°C. Then, protein samples from the control group were labeled with reporter tags 114 and 116 and protein samples from the YDH model group were labeled with reporter tags 113 and 115. The labeled peptides were pooled and desalted and then dried by a rotation vacuum concentrator (Christ RVC 2-25, Christ, Osterode, Germany).

The iTRAQ-labeled peptides were fractionated by strong cation exchange (SCX) liquid chromatography using a polysulfoethyl column (2.1 × 100 mm, 5 *μ*m, 200 Ǻ; Nest Group, Inc., Southborough, MA, USA). A gradient of buffer A (10 mM KH_2_PO_4_, 25% ACN, pH 2.6) and buffer B (350 mM KCl, 10 mM KH_2_PO_4_, 25% ACN, pH 2.6) was used to elute the peptides adsorbed on the column. The concentrations of the eluted peptides were monitored by measuring the absorbance at 214 nm [10]. A total of ten SCX fractions were collected and concentrated. Each fraction was dissolved and subjected to Reverse-phase LC fractionation. The LC elution was subsequently analyzed on a Triple TOF 5600 system (Applied Biosystems) in duplicate as two technical replicates. In an information-dependent acquisition mode (IDA), the survey scans were acquired from 400 to 1500* m/z* with up to 20 most intense multiply charged ions selected for MS/MS analysis [11]. The product ion spectra were accumulated in the 100–2000* m/z* to enhance the intensities of the iTRAQ reporter ions (113, 114, 115, and 116* m/z*) for quantification. The relative abundance of the peptides and proteins was reflected by the ratio of the peak area of the iTRAQ reporter ions intensities [12].

The data of MS/MS spectra were searched against the IPI rat database (IPI.rat.v3.69, 39 578 entries) with ProteinPilot™ 2.0.1 software (Applied Biosystems) [13]. ProteinPilot was searched with a fragment ion mass tolerance of 0.050 Da and a parent ion tolerance of 10.0 ppm. Scaffold (version Scaffold_4.6.2, Proteome Software Inc., Portland, OR) was used to validate MS/MS based peptide and protein identifications. Peptide identifications were accepted if they could establish an FDR less than 1.0% by the Scaffold Local FDR algorithm and contained at least 2 identified peptides. Proteins that contained similar peptides and could not be differentiated based on MS/MS analysis alone were grouped to satisfy the principles of parsimony. Scaffold Q+ (version Scaffold_4.6.2, Proteome Software Inc., Portland, OR) was used to quantitate Label Based Quantitation (iTRAQ, TMT, SILAC, etc.) peptide and protein identifications. Normalization was performed iteratively (across samples and spectra) on intensities, as described in Statistical Analysis of Relative Labeled Mass Spectrometry Data from Complex Samples Using ANOVA [14]. Medians were used for averaging. Spectra data were log-transformed, pruned of those matched to multiple proteins, and weighted by an adaptive intensity weighting algorithm. *p* values less than 0.05 in each independent iTRAQ experiment and the fold-changes ratio lower than 0.8 or higher than 1.25 were considered as significant [15, 16].

### 2.4. Bioinformatics Analysis

We annotated function or feature of proteins from several different categories, including Gene Ontology (GO), Kyoto Encyclopedia of Genes and Genomes (KEGG) pathway, and Search Tool for the Retrieval of Interacting Genes/Proteins (STRING) software. The molecular function, cellular component, and biological process were analyzed by searching GO database (http://geneontology.org/). The signal pathways of proteins were annotated by KEGG pathway database (http://www.genome.jp/kegg/mapper.html). The interaction networks of the identified proteins were analyzed by STRING software (http://string-db.org/).

### 2.5. Enzyme-Linked Immunosorbent Assay (ELISA)

Kininogen 1 (KNG1) Rat ELISA Kit (Abcam, detection limit 4.97 ng/mL), Apoc3 (Rat/Mouse) ELISA Kit (Abnova, detection limit 0.3 g/mL), and rat paraoxonase 1 (PON1) ELISA Kit (Cusabio Biotech, Wuhan, Hubei, China; detection limit 15.6 U/mL) were used to measure the expression levels of proteins. Briefly, serum samples were incubated in microtiter wells with appropriate dilution according to the manufacturer's instructions. After discarding the solution and washing, biotinylated antibody, HRP-streptavidin, substrate reagent, and stop solution were sequentially added to the wells. Finally, absorbance was read at wavelength of 450 nm immediately. The expression levels of the proteins were calculated by a four-parameter logistic curve (Microplate manager 6 software, Bio-Rad).

### 2.6. Statistical Analysis

Experimental data were presented as mean ± standard deviation (SD). Statistical analyses were performed with SPSS software, version 18.0 (SPSS, Chicago, IL, USA) and *p* value of less than 0.05 was considered statistically significant. Nonparametric analyses were performed using the Mann–Whitney *U* test. Receiver operating characteristic (ROC) curves and logistic regression models were performed using MedCalc Software (Version 12.4.2.0, Belgium).

## 3. Results

### 3.1. Characteristics of YDH Syndrome Rats

**Figure 2:**
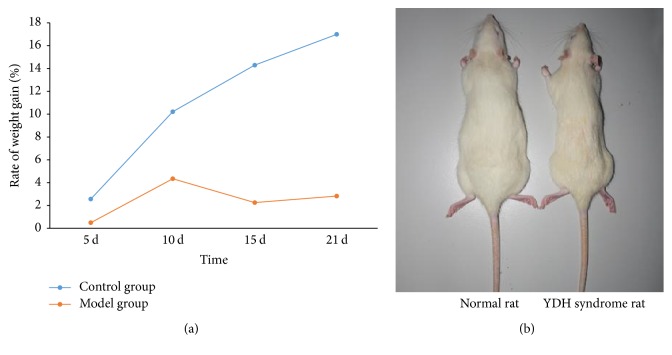
(a) The rate of weight gain in the normal rats and the YDH syndrome rats at each time point. (b) Gross appearance of the YDH syndrome rat compared to the normal rat at the end of the experiment.

**Figure 3:**
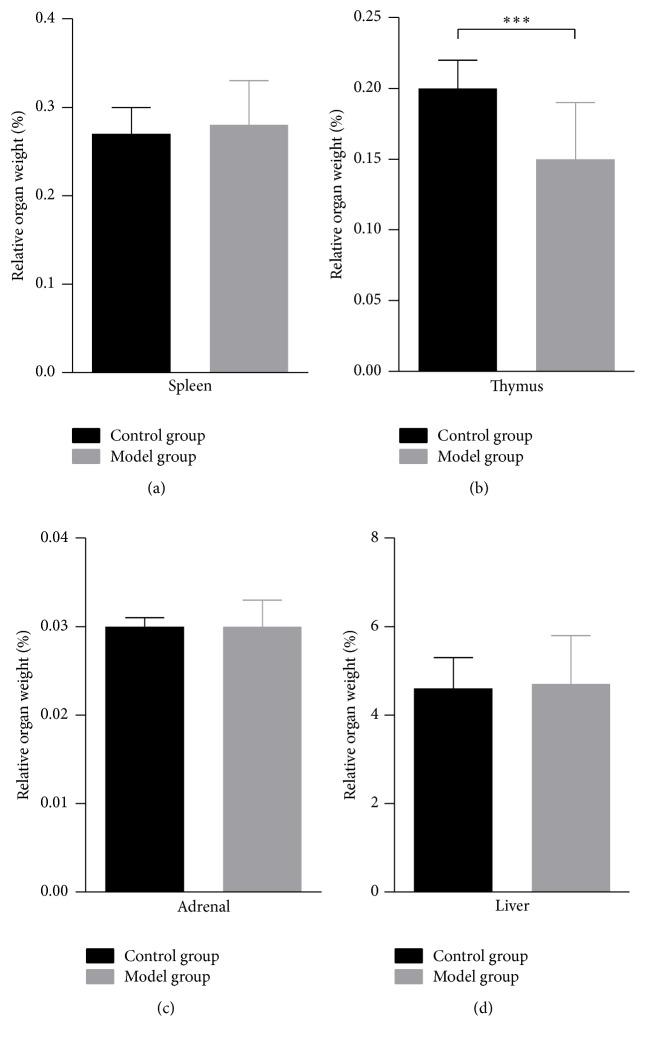
The comparison of relative organs weight between the normal rats and YDH syndrome rats. Values are means ± SD. *p* value of less than 0.05 indicates statistical significance using the Mann–Whitney *U* test. ^*∗∗∗*^ *p* < 0.001. (a) The relative weight of the spleen in each group. (b) The relative weight of the thymus in each group. (c) The relative weight of the adrenal gland in each group. (d) The relative weight of the liver in each group.

Compared with the normal group, rats in the model group showed obvious YDH symptoms, including dry hair, restlessness, dry stool, and weight loss. The rate of weight gain in the YDH syndrome rats was consistently lower than that of the control group (Figure 2(a)). At the end of the third week, rats in the YDH model group appeared much smaller than the normal rats (Figure 2(b)), and the relative thymus weight decreased significantly in the model group compared with the normal group (*p* < 0.001, Figure 3(b)). However, the relative weights of the spleen, adrenal gland, and liver showed no remarkable changes (Figures 3(a), 3(c), and 3(d)).

### 3.2. Proteomic and Bioinformatics Analysis

**Figure 4:**
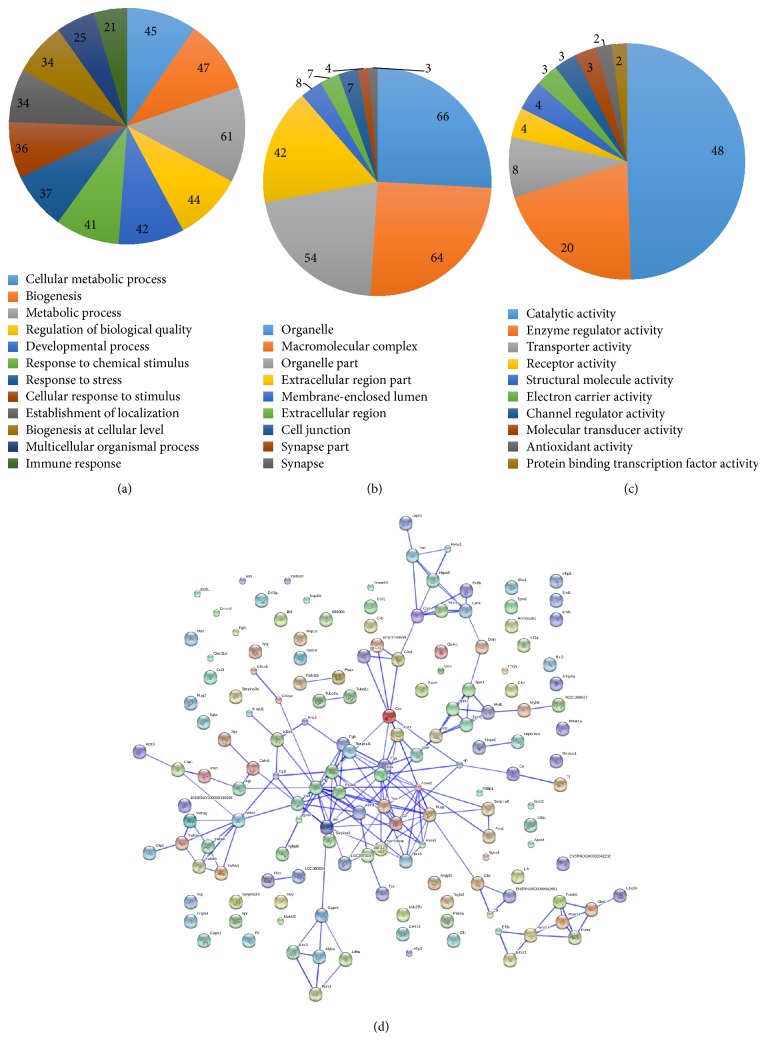
Data mining of the set of differentially expressed proteins in YDH syndrome rats. (a) Biological process. (b) Cellular component. (c) Molecular function. (d) The interacted network of proteins was analyzed by STRING (Search Tool for the Retrieval of Interacting Genes/Proteins) software.

The intensity distribution for all channels was shown in Supplementary Figure  1 in Supplementary Material available online at http://dx.doi.org/10.1155/2016/5176731, and higher values represent better signal intensities. The deviation of the MS data was less than 2.5 ppm, indicating a high degree of accuracy (Supplementary Figure  2). The distribution of discriminant score calculated by Scaffold software in PeptideProphet algorithm was shown in Supplementary Figure  3, and the channel signals before and after the normalization of the channel distribution were shown in Supplementary Figure  4. A total of 165 differentially expressed proteins were identified in the serum of YDH syndrome rats using iTRAQ-2D LC-MS/MS, 127, and 130 proteins in two replicates, respectively. 92 differentially expressed proteins were screened in both replicates, including 63 upregulated proteins (>1.25-fold, *p* < 0.05, Table 1) and 29 downregulated proteins (<0.8-fold, *p* < 0.05, Table 2). GO annotation was used to analyze the cellular component, molecular function, and participation in biological processes of these 92 distinct proteins. In the biological processes analysis, we found that a majority of differentially expressed proteins were associated with metabolic process (48, 36%; Figure 4(a)), indicating that metabolic abnormalities were closely related to the YDH syndrome. Cellular component analysis revealed that the differentially expressed proteins were mainly localized to the organelle (66, 26%), macromolecular complex (64, 25%), organelle part (54, 21%), and extracellular region part (42, 16%; Figure 4(b)). Function-based enrichment analysis indicated that most of these proteins played important roles in catalyzing (48, 50%) and enzyme regulating (20, 21%; Figure 4(c)). KEGG pathway annotation revealed a greater relative abundance of proteins involved in immune system (especially in complement and coagulation cascades, Table 3) and carbohydrate metabolism (especially in glycolysis and gluconeogenesis, Table 4), indicating that the YDH syndrome was closely related to the abnormality of immunoreaction and glycometabolism. Furthermore, STRING analysis showed that most of the differentially expressed proteins interacted with each other (Figure 4(d)).

### 3.3. Validation of Proteins by ELISA

**Figure 5:**
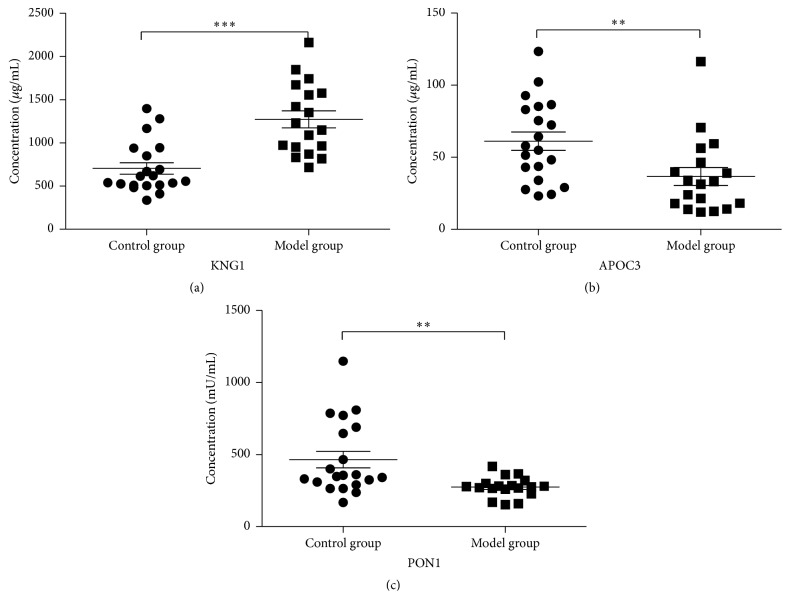
Validation of KNG, APOC3, and PON1 in serum. Levels of these proteins were measured by ELISA in serum of the control group (*n* = 20) and the model group (*n* = 18). Median values are shown by a horizontal line. *p* values were calculated with the Mann–Whitney *U* test. ^*∗∗∗*^ *p* < 0.001; ^*∗∗*^ *p* < 0.01.

According to the results of the bioinformatics analysis, several abnormally expressed proteins (KNG1, APOC3, and PON1) were validated by ELISA. The results showed that the serum levels of KNG1 in YDH syndrome rats (1271.80 ± 413.65 *μ*g/mL) were significantly higher (*p* < 0.0001) than that in normal rats (702.89 ± 296.43 *μ*g/mL) (Figure 5(a)). The serum levels of APOC3 in YDH syndrome rats and normal rats were 36.60 ± 26.43 *μ*g/mL and 61.04 ± 28.32 *μ*g/mL, respectively (Figure 5(b)), and the serum levels of PON1 in YDH syndrome rats and normal rats were 274.76 ± 69.23 mU/mL and 465.73 ± 254.91 mU/mL, respectively (Figure 5(c)). These data indicated that the levels of APOC3 (*p* < 0.005) and PON1 (*p* < 0.001) were significantly decreased in YDH syndrome rats. The results were in line with the iTRAQ data.

### 3.4. ROC Analysis

The data of serum protein levels (18 YDH syndrome rats and 20 normal rats) were subjected to forward stepwise multivariate regression analysis. The results indicated that KNG1, APOC3, and PON1 were all included in the diagnostic model as follows:

(1) p=e−0.4702+0.005951∗KNG1−0.008572∗APOC3−0.01412∗PON11+e−0.4702+0.005951∗KNG1−0.008572∗APOC3−0.01412∗PON1.

**Figure 6:**
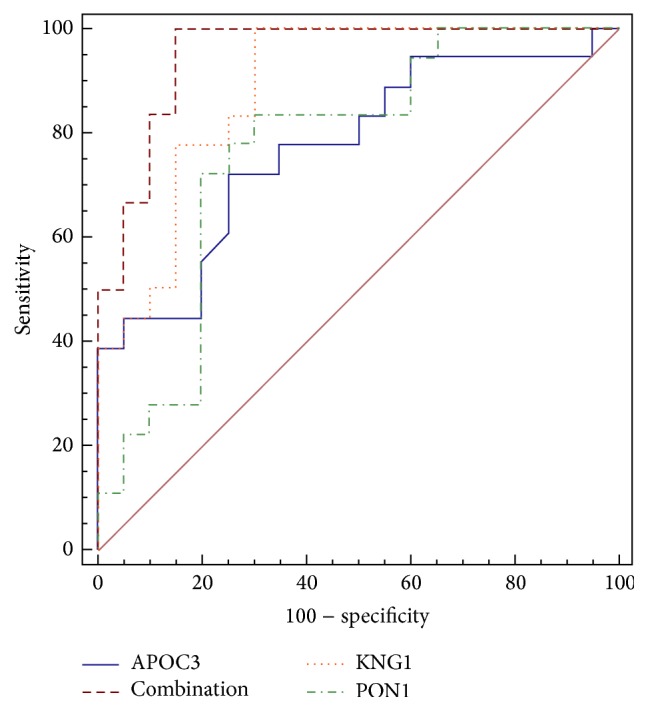
The receiver operation characteristics (ROC) curve analyses. ROC curve analyses of the serum protein KNG1, APOC3, and PON1 as well as the combination of the three proteins to discriminate YDH syndrome from controls.

Odds ratios for KNG1, APOC3, and PON1 in the model were 1.006, 0.991, and 0.986, respectively. The area under the ROC curve (AUC) in the diagnostic model was 0.950 (95% CI, 0.826–0.994, *p* < 0.0001, cutoff value was 0.3154). It was much higher than the AUC of KNG1 (0.886, 95% Cl, 0.741–0.966, *p* < 0.0001), APOC3 (0.771, 95% Cl, 0.606–0.891, *p* = 0.0006), and PON1 (0.767, 95% Cl, 0.601–0.888, *p* = 0.0009) alone (Figure 6). The sensitivity and specificity of KNG1, APOC3, and PON1 for detection of YDH syndrome were 100.0% and 70.0%, 72.2% and 75.0%, and 83.3% and 70.0%, respectively. The sensitivity and specificity of the diagnostic model (KNG1, APOC3, and PON1 combination) were 100.0% and 85.0%. In addition, the positive values and the negative predictive values for YDH syndrome diagnosis were also improved by the combination of KNG1, APOC3, and PON1 (Table 5).

## 4. Discussion

YDH syndrome is common in TCM practice and has been widely studied in recent decades. Many former studies have observed the abnormalities of immune function in YDH syndrome individuals. Wang found decline in immune function with a decrease of immunological substances in YDH constitution [17]. In addition, TNF-*α*, IL-1*β*, and IL-6 levels have been found to be elevated, while IL-2 levels have been found to be decreased in YDH syndrome individuals [18, 19]. Liu demonstrated decreased CD3+ and CD4+ levels in the peripheral blood of YDH syndrome individuals [20], indicating that the immune function is declined in YDH syndrome. Notably, we found that the weight of the thymus, one of the most important immune organs, was significantly decreased in YDH syndrome rats, suggesting reduced immune function in YDH syndrome. In iTRAQ-2DLC-MS/MS experiment, it was interesting to find that a majority of differentially expressed proteins were involved in immune response and metabolic processes. KEGG pathway analysis revealed that the immune response-associated proteins were mainly clustered at complement and coagulation cascades. To our knowledge, the deficiency of complement significantly reduces IL-1*β* levels [21, 22], and TNF-*α* contributes to coagulation and complement activation in virus-induced fulminant hepatitis [23], accounting for the upregulation of IL-1*β* and TNF-*α* levels in YDH syndrome individuals. Based on these findings, we speculated that the abnormally expressed proteins involved in complement and coagulation cascades disrupted the signaling pathways related to immune response. Accordingly, immune function in YDH syndrome was disturbed.

Previous studies have revealed that the activities of energy-yielding and energy-consuming reactions were enhanced in YDH syndrome rats [24], which was in line with our results of proteomic experiment. By comparing the differentially expressed serum proteins between YDH syndrome rats and normal rats, we found that approximately half of these proteins were associated with the metabolic processes. KEGG pathway analysis demonstrated that proteins participating in carbohydrate metabolism pathway, especially glycolysis and gluconeogenesis, accounted for a great proportion of the metabolism-associated proteins, which was consistent with previous studies revealing the lower levels of glycogen in YDH syndrome rats [24] and the hyperactivity of glycolysis in epithelial tissue with YDH syndrome [25]. Our results revealed that the enhanced energy metabolism in YDH syndrome attributed mostly to glucose metabolism, which might account for the weight loss in YDH syndrome individuals and animal models.

In the present study, we found that the level of KNG1 was significantly increased in the serum of YDH rats (*p* < 0.0001). KNG1, a precursor protein of vasoactive kinin [26], is known to participate in inflammation, coagulation, and innate immunity [27]. KNG1 has been found to be significantly elevated in uterine proliferative lesions rats [28] and the mRNA expression of the KNG1 gene has been shown to be upregulated in mouse lung tumors [29]. High-molecular-weight kininogen (HK) is cleaved by kallikrein to release bradykinin (BK) and HKa. HKa induces mononuclear cells to release cytokines (TNF- *α*, IL-1, and IL-6) and chemokines (IL-8 and MCP-1) [30]. Interestingly, it has been demonstrated previously that the levels of TNF-*α*, IL-1*β*, and IL-6 were higher in YDH syndrome individuals, suggesting that KNG1 is highly associated with the immune abnormalities in YDH syndrome.

PON1 is an important antioxidant enzyme against oxidative stress [31], which protects low-density lipoprotein (LDL) against oxidative processes [32] and prevents the formation of oxidized- (Ox-) LDL [33]. Ox-LDL facilitates the proliferation of endothelial cells and the accumulation of macrophages in arterial wall [34] and has been shown to be associated with high oxidative stress [35, 36]. So, PON1 may reduce the oxidative stress in the body. In this study, we found that the serum level of PON1 declined considerably in YDH syndrome rats (*p* = 0.0052), which was consistent with the PON1 alterations in cardiovascular disorders, cancer, and acute influenza infection [37, 38]. YDH syndrome, induced by emotional stress or long-term intake of spicy and hot food, is characterized by mucosal lesions. Meanwhile, oxidative stress plays an important role in the pathogenesis of mucosal lesions [39]. Therefore, the low levels of PON1 may indirectly increase oxidative stress in YDH individuals, subsequently contributing to the mucosal lesions.

APOC3 is closely correlated to lipid metabolism. It is an inhibitor of hepatic lipase and lipoprotein lipase [40], which interferes with lipolysis. Maeda et al. found that the plasma triglyceride levels in mice lacking gene APOC3 reduced to approximately 70% of normal level [41]. Another study indicated that the overexpression of APOC3 can lead to hypertriglyceridemia* in vivo* [42]. In the present study, obvious weight loss was observed in YDH syndrome rats, and the rate of weight gain was consistently lower than normal rats. Serum APOC3 levels in YDH syndrome rats decreased significantly (*p* = 0.0046). So, we suggested that rats with lower APOC3 have higher activities of hepatic lipase and lipoprotein lipase and undergo more adipose tissue lipolysis compared with normal rats.

## 5. Conclusion

In this study, we found that YDH syndrome is highly associated with the disturbance in immune response and metabolic processes. Further proteomic analysis revealed that YDH syndrome was mainly attributed to the disruption in complement and coagulation pathway and the glucose metabolism pathway. Besides, we revealed that the increased serum KNG1 levels and the decreased serum APOC3 and PON1 levels were closely related to YDH syndrome. KNG1 may regulate cytokines and chemokines release during the abnormal immune responses in YDH syndrome, while the low levels of PON1 and APOC3 may increase oxidative stress and lipolysis in YDH syndrome, respectively. The results indicated that KNG1, APOC3, and PON1 may act as potential biomarkers for the diagnosis of YDH syndrome. To accelerate the process of applying our findings in clinical practice, further study to evaluate the effectiveness in a relatively large sample size in YDH syndrome patients is needed. This study provides a novel method to diagnose YDH syndrome and may have significant implications for revealing the molecular mechanism of YDH syndrome.

## Supplementary Material

The quality control reports of iTRAQ-2D LC-MS/MS experiments

## Figures and Tables

**Table 1:** Upregulated serum proteins in YDH syndrome group quantified by iTRAQ-based proteomics and the ratios to the control group.

Protein ID	Gene name	Protein name	M/C (114/113)			M/C (116/115)		
			Run 1	Run 2	Mean	Run 1	Run 2	Mean
P62161	Calm1	Calmodulin	8.09	16.75	12.42	12.82	6.08	9.45
P62260	Ywhae	14-3-3 protein epsilon	2.68	6.31	4.49	15.28	14.59	14.93
Q6AYZ1	Tuba1c	Tubulin alpha-1C chain	9.29	4.79	7.04	3.91	17.22	10.56
P45592	Cfl1	Cofilin-1	4.37	3.44	3.90	11.07	13.93	12.50
P01048	Map1	T-kininogen 1	5.50	4.57	5.03	16.29	5.65	10.97
P09006	Serpina3n	Serine protease inhibitor A3N	2.99	2.86	2.92	11.91	13.18	12.55
P08932	—	T-kininogen 2	3.91	4.79	4.35	14.72	6.14	10.43
Q62812	Myh9	Myosin-9	7.11	9.55	8.33	6.79	6.03	6.41
P18418	Calr	Calreticulin	4.13	2.75	3.44	8.55	9.73	9.14
Q66HD0	Hsp90b1	Endoplasmin	3.02	6.25	4.64	13.55	2.03	7.79
P34058	Hsp90ab1	Heat shock protein HSP 90-beta	4.25	9.64	6.94	5.25	5.50	5.37
P68511	Ywhah	14-3-3 protein eta	3.84	1.46	2.65	13.68	5.06	9.37
P04276	Gc	Vitamin D-binding protein	6.61	4.45	5.53	5.65	6.03	5.84
Q62636	Rap1b	Ras-related protein Rap-1b	3.19	2.58	2.89	8.71	7.66	8.18
Q9Z1P2	Actn1	Alpha-actinin-1	2.99	2.63	2.81	6.79	8.79	7.79
P09495	Tpm4	Tropomyosin alpha-4 chain	2.81	2.51	2.66	6.14	9.12	7.63
Q6PCT3	Tpd52l2	Tumor protein D54	1.71	6.31	4.01	4.41	7.87	6.14
P11980	Pkm	Pyruvate kinase isozymes M1/M2	1.82	2.56	2.19	11.27	4.57	7.92
Q08163	Cap1	Adenylyl cyclase-associated protein 1	3.22	3.94	3.58	4.17	8.39	6.28
P11598	Pdia3	Protein disulfide-isomerase A3	11.17	3.16	7.17	2.13	2.27	2.20
P16086	Sptan1	Spectrin alpha chain	2.38	3.16	2.77	5.92	6.92	6.42
P62963	Pfn1	Profilin-1	3.13	2.31	2.72	4.79	7.45	6.12
Q68FR2	Bin2	Bridging integrator 2	2.25	2.17	2.21	6.03	6.08	6.05
Q63610	Tpm3	Tropomyosin alpha-3 chain	2.21	2.70	2.46	5.30	6.25	5.77
P06866	Hp	Haptoglobin	7.11	3.50	5.31	2.75	3.05	2.90
P08934	Kng1	Kininogen-1	5.86	4.88	5.37	1.60	2.83	2.22
B0BNA5	Cotl1	Coactosin-like protein	2.56	2.25	2.40	5.65	4.53	5.09
Q63081	Pdia6	Protein disulfide-isomerase A6	3.02	4.21	3.61	4.29	3.44	3.86
P85972	Vcl	Vinculin	3.56	3.08	3.32	4.09	3.94	4.02
P04642	Ldha	L-lactate dehydrogenase A chain	4.41	1.67	3.04	6.61	1.82	4.21
P63102	Msfs1	14-3-3 protein zeta/delta	2.81	1.84	2.32	5.20	4.53	4.86
B2GUZ5	Capza1	F-actin-capping protein subunit alpha-1	2.23	1.72	1.98	3.73	6.49	5.11
P46462	Vcp	Transitional endoplasmic reticulum ATPase	2.99	2.40	2.70	2.65	5.45	4.05
P20767	—	Ig lambda-2 chain C region	2.25	2.05	2.15	2.58	6.55	4.56
Q5XFX0	Tagln2	Transgelin-2	3.63	2.07	2.85	2.81	4.37	3.59
Q4V7E8	Lrrfip2	Flightless-interacting protein 2	2.33	1.96	2.15	4.06	3.98	4.02
Q63617	Hyou1	Hypoxia upregulated protein 1	1.79	5.97	3.88	1.75	2.58	2.17
Q62930	C9	Complement component C9	3.63	2.83	3.23	2.25	3.25	2.75
Q5XI73	Arhgdia	Rho GDP-dissociation inhibitor 1	1.74	2.99	2.37	2.96	4.02	3.49
P04797	Gapdh	Dehydrogenase	1.77	1.51	1.64	3.50	4.92	4.21
Q07009	Capn2	Calpain-2 catalytic subunit	1.28	3.60	2.44	3.80	2.65	3.23
Q5U211	Snx3	Sorting nexin-3	1.91	4.25	3.08	1.60	3.22	2.41
Q63514	C4bpa	C4b-binding protein alpha chain	2.99	2.49	2.74	2.88	2.31	2.60
Q64119	Myl6	Myosin light polypeptide 6	1.72	2.27	2.00	3.60	2.68	3.14
P97571	Capn1	Calpain-1 catalytic	3.73	2.15	2.94	2.11	1.61	1.86
P20760	Igg-2a	Ig gamma-2A chain C region	1.50	1.84	1.67	2.63	3.22	2.93
P06761	Hspa5	78 kDa glucose-regulated protein	1.56	1.46	1.51	3.53	2.56	3.05
Q63515	C4bpb	C4b-binding protein beta chain	3.60	2.09	2.84	1.56	1.61	1.59
Q7M0E3	Dstn	Destrin	3.16	1.29	2.23	2.61	1.58	2.10
Q9EPH8	Pabpc1	Polyadenylate-binding protein 1	1.41	1.80	1.60	3.40	2.00	2.70
P63018	Hspa8	Heat shock cognate 71 kDa protein	1.60	2.19	1.89	2.63	2.17	2.40
P04785	P4hb	Protein disulfide-isomerase	2.25	2.01	2.13	1.82	2.49	2.15
Q9EQX9	Ube2n	Ubiquitin-conjugating enzyme E2 N	2.86	1.58	2.22	1.87	2.15	2.01
P06399	Fga	Fibrinogen alpha chain	4.37	1.43	2.90	1.33	1.28	1.31
P01835	—	Ig kappa chain C region	2.01	1.57	1.79	2.03	2.70	2.37
P12346	Tf	Serotransferrin	1.60	1.91	1.75	2.54	2.03	2.28
Q9JLT6	Bid	BH3-interacting domain death agonist	1.36	1.64	1.50	1.69	3.05	2.37
P68101	Eif2s1	Eukaryotic translation initiation factor	1.66	1.26	1.46	3.16	1.46	2.31
P35213	Ywhab	14-3-3 protein beta/alpha	1.85	1.28	1.57	2.65	1.49	2.07
Q91ZN1	Coro1a	Coronin-1A	1.64	1.37	1.51	2.36	1.69	2.02
P04906	Gstp1	Glutathione S-transferase P	1.33	1.47	1.40	2.51	1.67	2.09
P14272	Klkb1	Plasma kallikrein	2.11	1.96	2.03	1.27	1.42	1.34
P07335	Ckb	Creatine kinase B-type	1.41	1.28	1.34	1.57	1.38	1.48

M, model group; C, control group; 114/113 and 116/115, two biological replicates; Run 1 and Run 2, two technical replicates.

**Table 2:** Downregulated serum proteins in YDH syndrome group quantified by iTRAQ-based proteomics and the ratios to the control group.

Protein ID	Gene name	Protein name	M/C (114/113)			M/C (116/115)		
			Run 1	Run 2	Mean	Run 1	Run 2	Mean
P02091	Hbb	Hemoglobin subunit beta-1	0.71	0.70	0.71	0.67	0.70	0.68
P05545	Serpina3k	Serine protease inhibitor A3K	0.66	0.64	0.65	0.65	0.70	0.68
Q6IE52	Mug2	Murinoglobulin-2	0.67	0.75	0.71	0.42	0.73	0.58
P15978	RT1-Aw2	Class I histocompatibility antigen	0.73	0.61	0.67	0.58	0.60	0.59
P36953	Afm	Afamin	0.54	0.45	0.49	0.74	0.69	0.72
Q62894	Ecm1	Extracellular matrix protein 1	0.77	0.55	0.66	0.52	0.51	0.52
O88201	Clec11a	C-type lectin domain family 11 member A	0.77	0.53	0.65	0.32	0.72	0.52
P55314	C8b	Complement component C8 beta chain	0.74	0.74	0.74	0.37	0.47	0.42
P14630	Apom	Apolipoprotein M	0.64	0.44	0.54	0.53	0.60	0.57
P35859	Igfals	Insulin-like growth facto	0.50	0.58	0.54	0.44	0.58	0.51
P14046	A1i3	Alpha-1-inhibitor 3	0.48	0.56	0.52	0.67	0.34	0.50
Q64268	Serpind1	Heparin cofactor 2	0.74	0.70	0.72	0.24	0.31	0.27
Q68FP1	Gsn	Gelsolin	0.56	0.58	0.57	0.31	0.52	0.41
Q00918	Ltbp1	Latent-transforming growth factor	0.44	0.50	0.47	0.44	0.47	0.46
P23680	Apcs	Serum amyloid P-component	0.73	0.69	0.71	0.22	0.21	0.22
Q8R2H5	Gpld1	Phosphatidylinositol	0.53	0.69	0.61	0.30	0.31	0.31
P31211	Serpina6	Corticosteroid-binding globulin	0.54	0.50	0.52	0.36	0.36	0.36
Q9QUH3	Apoa5	Apolipoprotein A-V	0.53	0.53	0.53	0.39	0.28	0.33
P48199	Crp	C-reactive protein	0.72	0.72	0.72	0.10	0.19	0.14
O70535	Lifr	Leukemia inhibitory factor receptor	0.33	0.40	0.36	0.46	0.52	0.49
P55797	Apoc4	Apolipoprotein C-IV	0.33	0.53	0.43	0.43	0.41	0.42
P18292	F2	Prothrombin	0.65	0.64	0.65	0.13	0.25	0.19
P07092	Serpine2	Glia-derived nexin	0.52	0.37	0.44	0.42	0.14	0.28
P19939	Apoc1	Apolipoprotein C-I	0.34	0.35	0.34	0.32	0.34	0.33
P07808	Npy	Pro-neuropeptide Y	0.63	0.29	0.46	0.13	0.24	0.19
O08770	Gp5	Platelet glycoprotein V	0.37	0.56	0.46	0.17	0.14	0.15
P55159	Pon1	Serum paraoxonase/arylesterase 1	0.27	0.19	0.23	0.31	0.24	0.28
P06759	Apoc3	Apolipoprotein C-III	0.41	0.28	0.35	0.13	0.11	0.12
P04638	Apoa2	Apolipoprotein A-II	0.11	0.13	0.12	0.25	0.27	0.26

M, model group; C, control group. 114/113 and 116/115, two biological replicates; Run 1 and Run 2, two technical replicates.

**Table 3:** KEGG pathways analysis of the differentially expressed proteins categorized by organismal systems.

Pathway	Number of matched proteins	Organismal systems	Pathway map
Complement and coagulation cascades	9	Immune system	rno04610
Antigen processing and presentation	7	Immune system	rno04612
Leukocyte transendothelial migration	5	Immune system	rno04670
Fc gamma R-mediated phagocytosis	4	Immune system	rno04666
NOD-like receptor signaling pathway	2	Immune system	rno04621
B cell receptor signaling pathway	1	Immune system	rno04662
Natural killer cell mediated cytotoxicity	1	Immune system	rno04650
Intestinal immune network for IgA production	1	Immune system	rno04672
Chemokine signaling pathway	1	Immune system	rno04062
Fc epsilon RI signaling pathway	1	Immune system	rno04664
Hematopoietic cell lineage	1	Immune system	rno04640
Neurotrophin signaling pathway	8	Nervous system	rno04722
Long-term potentiation	1	Nervous system	rno04720
Vasopressin-regulated water reabsorption	2	Excretory system	rno04962
Endocrine	1	Excretory system	rno04961
Circadian rhythm - mammal	1	Environmental adaptation	rno04710
Insulin signaling pathway	3	Endocrine system	rno04910
Progesterone-mediated oocyte maturation	1	Endocrine system	rno04914
Renin-angiotensin system	1	Endocrine system	rno04614
PPAR signaling pathway	2	Endocrine system	rno03320
Mineral absorption	1	Digestive system	rno04978
Pancreatic secretion	2	Digestive system	rno04972
Axon guidance	1	Development	rno04360
Cardiac muscle contraction	4	Circulatory system	rno04260

**Table 4:** KEGG pathways analysis of the differentially expressed proteins categorized by metabolism.

KEGG categories	Number of matched proteins	Metabolism	Pathway map
Glycolysis/gluconeogenesis	4	Carbohydrate metabolism	rno00010
Pyruvate metabolism	2	Carbohydrate metabolism	rno00620
Starch and sucrose metabolism	1	Carbohydrate metabolism	rno00500
Fructose and mannose metabolism	1	Carbohydrate metabolism	rno00051
Propanoate metabolism	1	Carbohydrate metabolism	rno00640
Pentose phosphate pathway	1	Carbohydrate metabolism	rno00030
Glycosylphosphatidylinositol- (GP-I) anchor biosynthesis	1	Glycan biosynthesis and metabolism	rno00563
Arginine and proline metabolism	1	Amino acid metabolism	rno00330
Drug metabolism, cytochrome P450	1	Xenobiotics biodegradation and metabolism	rno00982
Folate biosynthesis	1	Metabolism of cofactors and vitamins	rno00790
Glutathione metabolism	1	Metabolism of other amino acids	rno00480
Purine metabolism	2	Nucleotide metabolism	rno00230
Metabolism of xenobiotics by cytochrome P450	1	Xenobiotics biodegradation and metabolism	rno00980
Cysteine and methionine metabolism	1	Amino acid metabolism	rno00270
Valine, leucine, and isoleucine biosynthesis	1	Amino acid metabolism	rno00290

**Table 5:** Diagnostic value for YDH syndrome detection of the individual markers and KNG1, APOC3, and PON1 combination.

Protein	Sensitivity (%)	Specificity (%)	PPV (%)	NPV (%)	AUC	95% CI	*p *value
KNG1	100.0	70.0	75.0	100.0	0.886	0.741–0.966	*p* < 0.0001
APOC3	72.0	75.0	72.2	75.0	0.771	0.606–0.891	*p* = 0.0006
PON1	83.3	70.0	71.4	82.4	0.767	0.601–0.888	*p* = 0.0009
KNG1 + APOC3 + PON1	100.0	85.0	85.7	100.0	0.950	0.826–0.994	*p* < 0.0001

AUC, area under the curve; PPV, positive predictive values; NPV, negative predictive values; 95% CI, 95% confidence interval.
