# Supplementary material for: Serum Protein KNG1, APOC3, and PON1 as Potential Biomarkers for Yin-Deficiency-Heat Syndrome
Source: Evid Based Complement Alternat Med. 2016 Oct 24;2016:5176731. doi: 10.1155/2016/5176731 (PMC5098100; doi:10.1155/2016/5176731)
Supplement: Supplementary file 1 — The quality control reports of iTRAQ-2D LC-MS/MS experiments [file 5176731.f1.docx]

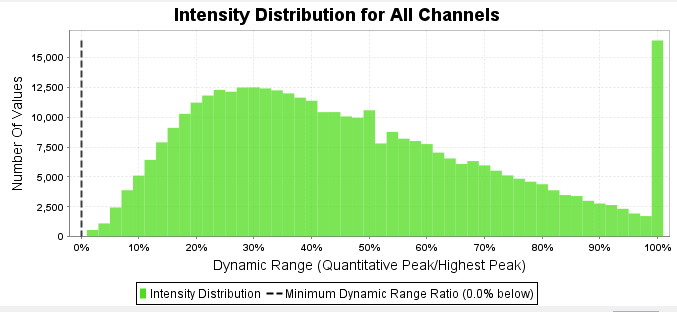


**Supplementary figure 1. The intensity distribution for all channels.** The X-axis indicates the ratio of the channel intensity and the strongest ion intensity in the same channel. Higher values represents better signal intensities.

Rep1


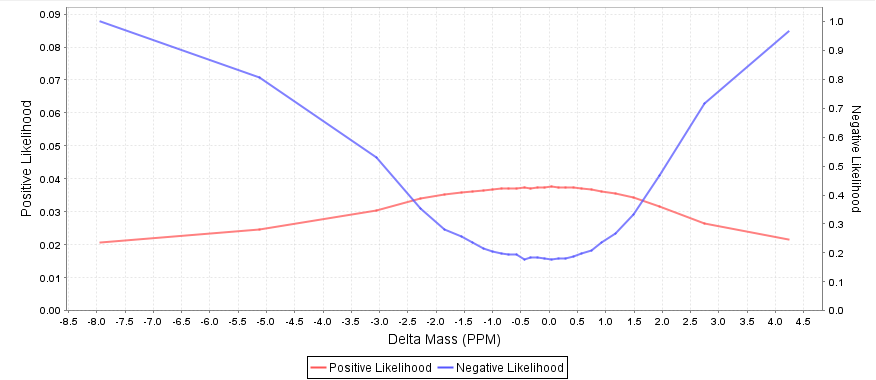


Rep2


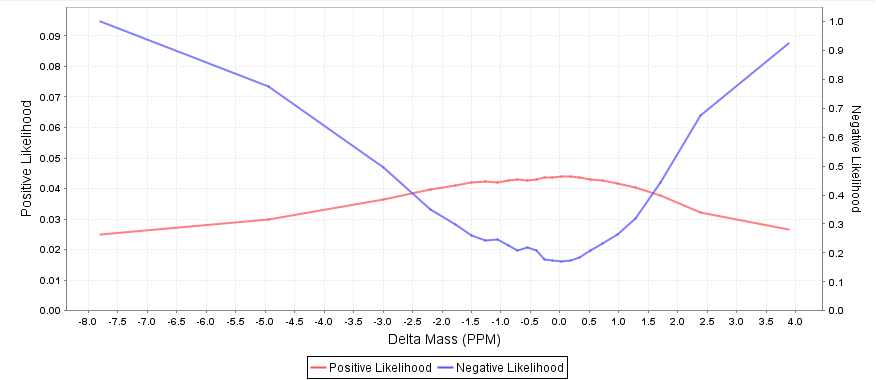


**Supplementary figure 2. Mass spectrometry accuracy assessment.** Generally, the range of accuracy is ±5 ppm, and the tolerance of accuracy is 10.0 ppm. The red curve and the blue curve represent the deviation distribution of positive results and negative results. The intersection of negative curve and positive curve indicates the accuracy of MS. In this study, the accuracy was 2.5 ppm, indicating the MS results were stable and reliable.

Rep1


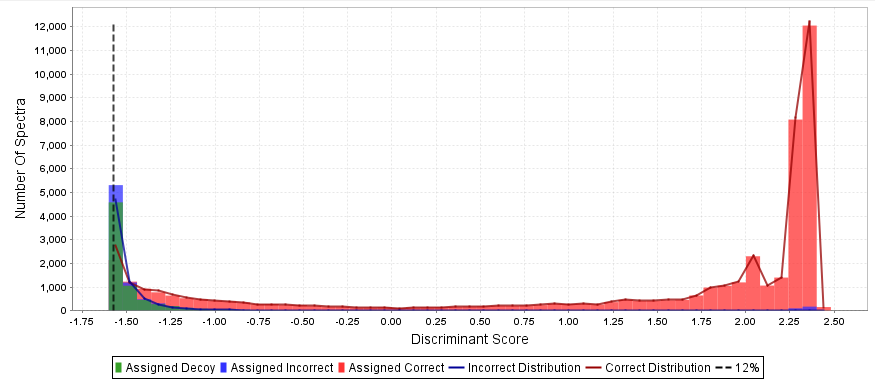


Rep2


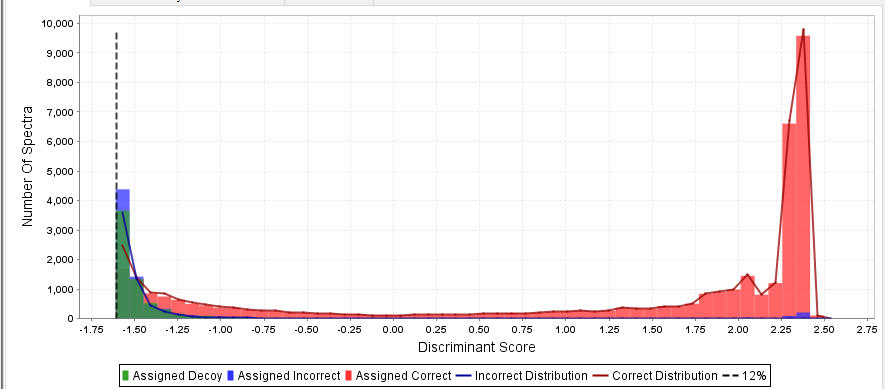


**Supplementary figure 3. The distribution of discriminant score calculated by Scaffold software in PeptideProphet algorithm.** Green area indicates assigned decoy data, blue area indicates incorrect data, and red area indicates correct data. The dashed line denotes the confidence threshold of the peptide. The wider distance red area separate from green and blue area, and the bigger value of area under the red curve, suggest better results.


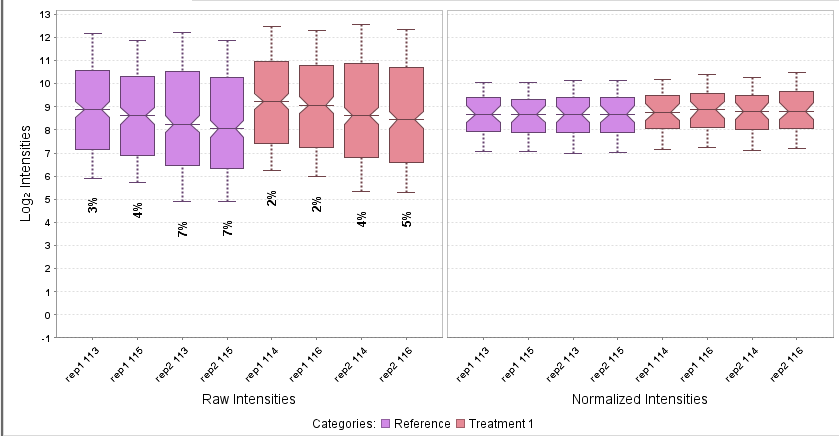


**Supplementary figure 4.** The channel signals before (left) and after (right) the normalization of the channel distribution. The percentages of missing ion in each channel are shown with a percent sign (%). The consistency of the median in each channel indicates the high uniformity of samples before mixing. The difference between the channel with highest median and the lowest median before normalization should less than 0.5, and the median should be in a horizontal line after normalization. In this study, the difference between the channel with highest median and the lowest median was 1, and the median in each channel were consistent after normalization.
